# Developing a topic-based repository of clinical trial individual patient data: experiences and lessons learned from a pilot project

**DOI:** 10.1186/s13643-021-01717-2

**Published:** 2021-06-01

**Authors:** Nancy Medley, Anna Cuthbert, Richard Crew, Lesley Stewart, Catrin Tudur Smith, Zarko Alfirevic

**Affiliations:** 1 Cochrane Pregnancy and Childbirth, University of Liverpool, Liverpool, UK; 2 Department of Health Data Science, University of Liverpool, Liverpool, UK; 3 Centre for Reviews and Dissemination, University of York, York, UK

**Keywords:** IPD, Individual patient data, Data sharing, Repository, Barriers

## Abstract

**Background:**

Building a dataset of individual participant data (IPD) for meta-analysis represents considerable research investment as well as collaboration across multiple institutions and researchers. Making arrangements to curate and share the dataset beyond the IPD meta-analysis project for which it was established, for reuse in future research projects, would maximise the value of this investment.

**Methods:**

Our aim was to establish the Cochrane repository for individual patient data from clinical trials in pregnancy and childbirth (CRIB) as an example of how an IPD repository could become part of Cochrane infrastructure. We believed that establishing CRIB under Cochrane auspices would engender trust and encourage trial investigators to share data, and at the same time position Cochrane to take steps towards expanding the number of reviews with IPD synthesis.

**Results:**

CRIB was designed as a web-based platform to receive, host and facilitate onward sharing of de-identified data. Development was not straightforward and we did not fully achieve our aim as intended. We describe the challenges encountered and suggest ways that future repositories might overcome these. In particular, securing the legal agreements required to facilitate data sharing proved to be the main barrier, being time-consuming and more complex than anticipated.

**Conclusions:**

We would recommend that researchers conducting IPD meta-analysis should consider discussing the option to transfer the curated IPD datasets to a repository at the end of the initial meta-analysis and this should be recognised within the data sharing agreements made with the original data contributors.

## Background

Individual patient data (IPD) meta-analyses are used increasingly in systematic reviews. Obtaining IPD from trials allows collection of unreported information, more detailed evaluation of trial integrity, and standardisation across trials, including covariate and outcome definitions. IPD permits more detailed and flexible analysis including time-to-event analysis and modelling individual-level variation in outcomes. A great deal of time, effort and resource is invested in assembling, checking and standardising the IPD across trials and the resultant IPD dataset is a valuable resource, with potential use beyond the project for which it was established. This is particularly apparent when the process of assembling the IPD dataset has added new information such as additional follow-up data that have not been previously analysed. This harmonised IPD dataset can be effectively considered as a new ‘study’ and considerable research effort is wasted when such data are not made available beyond the end of the IPD meta-analysis project.

In recent years, a number of data repositories and data-sharing platforms have been established to store, curate and share data from completed clinical trials. These focus on storing or providing access to IPD in its original format as recorded by each individual trial, and they are geared towards sharing data from individual trials rather than harmonised datasets from groups of trials addressing the same research question. The establishment of these resources is a welcome development. However, the format and coding of IPD is likely to differ between stored trials and will need to be re-coded for use in any new IPD meta-analysis or other research projects that wish to use a dataset, comprising multiple trials, addressing similar questions in its entirety. The new research team may still need to consult with the original trial investigators, to clarify issues, recode variables and obtain any required information that is missing from the repository materials. Restrictions on how repository data may be used, particularly having to analyse data within a secure repository space, may potentially limit their usefulness for some types of IPD meta-analysis projects. Having to repeat data checking and harmonisation processes for each new project is wasteful of prior research effort if trials have previously been included in an IPD meta-analysis—for both the new research team and the original trial investigators. Furthermore, existing repositories have thus far been much more successful in securing data from commercial trials than from academic trials.

There is therefore space to establish repositories that provide long-term storage and curation of the data collected during IPD meta-analyses, or other similar projects that bring together data across multiple studies, with an option to add relevant new trials as they are completed. A number of such topic-based repositories exist, but they mostly focus on reusing the data for ongoing research collaboration within the group that established the original IPD meta-analysis. This reuse builds on the trust established during the original IPD meta-analysis, whereby the original trial investigators who are the data owners are comfortable with the central team who manage the data repository making decision about reuse on their behalf (although they may also have a right of veto on inclusion in new projects).

We know from experience that trial investigators are often wary of sharing data and need reassurance about exactly how their data will be used and what for. This often forms the basis of the data sharing agreements (DSA) that are put in place between IPD meta-analysis teams and trial investigators. These DSAs generally stipulate that the IPD will be used only for the project in hand, used in accordance with its protocol and not shared beyond the project. They also often set out the nature of collaborating and the academic credit that will accrue from participation, such as authorship arrangements.

Establishing repositories of trial data seeded by IPD meta-analysis projects that aim to share data beyond the collaborative group would be in the public interest, and has already been shown to be supported by the research community [1]. We believed that doing so under the auspices of Cochrane, a worldwide trusted organisation that specialises in systematic review, could enhance the chances of success. We thought that academic clinical trial investigators might be more willing to share their data and to delegate authority to approve third party requests to access and use it, to Cochrane as opposed to other organisations. This trust could be built on Cochrane’s general reputation and on the fact that governance processes could build on the considerable topic and methodological expertise that resides within the Cochrane community. At the same time, building such repositories could facilitate expansion of IPD meta-analysis within Cochrane reviews, and over time support exciting possibilities for IPD network meta-analyses and living IPD reviews.

## Methods

The objective was to establish an online platform (CRIB—Cochrane repository for individual patient data from clinical trials in pregnancy and childbirth) to facilitate the sharing of IPD datasets under the auspices of the Cochrane collaboration, using the EPPPIC (Evaluating Progestogens for Prevention of Preterm birth International Collaborative) [2] IPD dataset as an exemplar, with a view to further expansion in the future. EPPPIC was set up to evaluate whether, and under what conditions, different forms of progestogen may be effective in preventing preterm births and associated neonatal complications. The EPPPIC project was conducted by a research team based at the University of York, endorsed and advised by an international Secretariat and funded by the Patient-Centered Outcomes Research Institute (PCORI).

**Fig. 1:**
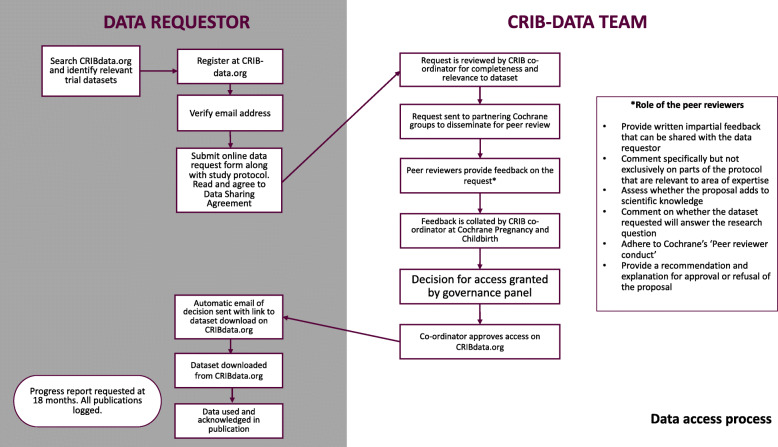
CRIB access process

To inform the development of CRIB, we examined the functionality of three major data sharing platforms (Vivli [3], UK Data Service [4], and Clinical Study Data Request (CSDR) [5]) as summarised in Table 1. Building on this, along with our personal experiences, recognition of FAIR Data principles [6], input from our advisory committee, and our previous work to elicit standards and preferences of the data sharing community [1, 7], we chose a ‘safeguarded’ data sharing model, where data users navigate an approvals process before being granted access to data. Our proposed data access process, including a Cochrane-affiliated peer review process and governance procedures are outlined in Fig. 1, and a brief comparison of CRIB features against other repositories is provided in Table 1. Development of CRIB, and the challenges therein, focussed on the following main areas:

**Table 1:** Data sharing platform comparison table

	CRIB	Vivli	UK data service	CSDR
Type of data access^1^	Safeguarded	Open, safeguarded, controlled	Open, safeguarded, controlled	Safeguarded
Functionality for controlled analyses within a secure platform	No	Yes	Yes	For pharmaceutical trials: Yes For non-commercial trials: No^2^
Formal assessment of disclosure risk before depositing data	No	No - Anonymisation services offered for a fee	Yes	Yes (advice on de-identification)
Trusted repository status (i.e. international standard ISO 16363)	No	Unclear	Yes	CSDR is not a repository; they signpost users to data owners
International standard for meta-data (e.g. DDI)	Not achieved	Yes	Yes	Unclear
International standard digital object identifier (DOI)	Not achieved	Yes	Yes	Yes
Who is the legal entity	University of Liverpool	Vivli	University of Essex	Agreement between data owner and data user
Who manages approvals	Cochrane pregnancy and childbirth	Data owner or Wellcome	UKDS	Data owner or Wellcome
Researcher training	No	No	Yes	No

^1^*Open data*, can be downloaded freely by anyone; *Safeguarded data*, downloadable but require an approvals process and legal agreements to structure data use (such as a licence agreement); *Controlled data*, pose disclosure risk to the organisation and can only be accessed by approved data users within a secured research environment (no download option and data use takes place in a secured setting)

^2^MRC, Cancer Research UK, Bill and Melinda Gates are piloting deposit of trial data with CSDR. Wellcome provide support and managed the independent review panel

## Results

### Platform

An online platform is required to manage requests, the approval process and data transfer between owners and users. A web-based application was developed so that changes in both governance and functionality could be easily accommodated. Given the nature of project, this approach proved to be useful as the requirements and functionality did indeed change during the lifespan of the exercise.

The approval process was developed to require a two-stage verification of the user’s request for data. Firstly, by simple verification of their email address and latterly an ‘offline check’ of the request by the CRIB team to establish the veracity and creditability of the requesting party.

The application itself has been developed using standard web technologies (principally Microsoft and Oracle) and hosted within the UoL data centre, in order to support the required level of security for a system such as this.

### Governance

Having had initial supportive conversations with members of the Cochrane Editorial and Methods Unit, our intention was that Cochrane would have an overarching responsibility for governance, and that the Pregnancy and Childbirth Group could be supported to manage the repository on a day to day basis. When explored in greater detail, the Cochrane legal department advised that they would not be willing to take the role of CRIB legal entity. This was a critical set-back as a main premise for the work was that entering into agreement with Cochrane would be a major incentive for trial investigators to share their data.

Cochrane proposed to fund the CRIB data being deposited through a recently established data sharing platform—Vivli [3]. Vivli presented Cochrane and CRIB with working drafts of both depositor and user agreements. We learned that Vivli’s $35,000 fee would not cover the costs of administrative support to manage secured data sharing. Another important point was that Vivli’s model included a fee for each individual trial in the dataset, even though the IPD derived dataset comprised all trials and could be treated as a single data set. The purpose of this pilot was to enable sharing of an IPD dataset comprising multiple trials.

In parallel, we explored the support offered by the UK Data Service [4], which included the opportunity to establish a dedicated ‘Cochrane collection’ that would host the EPPPIC IPD dataset under safeguarded management, along with an assessment of the risks of disclosure of the IPD dataset, Cochrane branding on the UKDS [4] website, and limited administrative support for vetting and sharing proposals to access data. The UKDS could assign the digital object identifier (DOI), create meta-data and had established legal agreements for data depositors that could be adopted and used.

### Legal

Whilst trying to create a feasible, user-friendly legal framework, we identified a few key obstacles. First, because the original EPPPIC data transfer agreements were developed and in use before CRIB was established, we had to seek additional permissions from the trial investigators to pass the data on to CRIB. Second, CRIB’s commitment to a model of safeguarded data sharing rather than open data sharing created requirements for additional legal agreements. The negotiations between legal teams from the University of York (EPPPIC), the University of Liverpool (CRIB) and Cochrane began early in the project, yet securing a workable legal framework for this model of data sharing was only been partially achieved after almost 3 years. An overview of the legal agreements required for the CRIB system is summarised in Table 2.

**Table 2:** The legal agreements required to facilitate proposed safeguarded data sharing through CRIB

Stakeholder	Document to sign
Trial data owners	• Data sharing agreement to create the IPD dataset • Data release form to permit transfer of cleaned, derived dataset to the repository • Depositor agreement with the Institution hosting the repository
Institution hosting the IPD data set (e.g. University)	Data transfer agreement
Institution hosting the repository (e.g. Cochrane)	Data transfer agreement
Approved data users	Research data access agreement with the Institution hosting the repository

### Recognition

Issues around assigning a DOI to the EPPPIC dataset, to enhance discoverability, were also problematic without backing from Cochrane as a legal entity, as the responsibility of assigning a DOI rests with an institution or organisation. As University of York (UoY) would not be the data host, it was not possible to issue a DOI. On the other hand, as UoY had a stake in the intellectual property through the value added by data harmonisation, University of Liverpool was not prepared to issue a DOI either. Consequently, no institution involved in our pilot project would agree to assign the EPPPIC/CRIB dataset a DOI, thus limiting the findability of the dataset, one of the key FAIR principles [6].

## Conclusions

Our experience confirms that many trial investigators support data sharing and reuse.

Our project intended to develop and pilot a mechanism for sharing a derived IPD dataset comprising multiple trials to facilitate reuse and maximise value from the research effort invested in conducting the individual trials and in establishing the harmonised IPD dataset. Our aim was not to compete with established platforms for sharing individual trials’ data, but to provide a framework for facilitating reuse of existing IPD datasets in future data synthesis, primarily by linking them to the relevant Cochrane review groups.

Despite the setbacks we encountered, we argue that Cochrane remains well positioned to lead the way in facilitating sharing trial data for use in IPD meta-analyses within systematic reviews. This would be contingent on reaching a shared view on sustainable hosting and maintenance of datasets. This would build on and enhance Cochrane’s reputation as an honest broker and global champion of ‘trusted evidence.’ A starting point for this would be to facilitate discussion within the Cochrane community about the value of such an endeavour. Hopefully, this process would increase pressure to find solutions to perceived legal stumbling blocks. At the same time, a detailed investigation of the various legal and data sharing agreements that need to be put in place, outside of the constraints of a time limited pilot project would be helpful, potentially including the development of a suite of generic legal agreements based on the templates from this pilot. Exploration of how DOIs for IPD meta-analysis datasets can be generated would also be valuable.

Finally, whilst we have demonstrated that the principles and technological side of establishing an online data sharing platform is feasible and relatively straightforward, we know that there are existing offerings available that could be adapted to suit the purpose intended. Subcontracting this aspect of the process carries a risk of diluting the value of Cochrane involvement as trial investigators would be entering into an agreement with external repositories. Nevertheless, with careful branding and transparency of involvement from Cochrane, this model could still be worthwhile. Our work has highlighted that some of these currently available offerings are costly and the important discussion of who should cover the financial commitment of sharing datasets of curated multiple trials, has only just started. We have identified the UK Data Service as a potentially suitable facility with several positive features that would be very worthy of further exploration.

Following further positive discussion with Cochrane, the current legal and contractual barriers to efficient use of IPD in systematic reviews have been acknowledged and Cochrane have confirmed they are committed to contributing to solutions. We believe that taking forward the discussions regarding hosting well-governed IPD repositories with Cochrane, or with another suitably trusted organisation, would reduce research waste, increase opportunities for IPD syntheses, and provide better evidence to inform decision-making.

## Data Availability

Not applicable
